# Impact of good governance, economic growth and universal health coverage on COVID-19 infection and case fatality rates in Africa

**DOI:** 10.1186/s12961-022-00932-0

**Published:** 2022-11-28

**Authors:** Bereket Gebremichael, Alemayehu Hailu, Mekitew Letebo, Etsub Berhanesilassie, Arega Shumetie, Sibhatu Biadgilign

**Affiliations:** 1 College of Health Science, Addis Ababa University, Addis Ababa, Ethiopia; 2 Department of Global Public Health and Primary Care, Bergen Center for Ethics and Priority Setting, University of Bergen, Bergen, Norway; 3 Harvard T.H. Chan School of Public Health, Harvard University, Boston, United States of America; 4 Independent Public Health Analyst and Research Consultant, P.O. BOX 24414, Addis Ababa, Ethiopia; 5 Ethiopian Economics Association, Addis Ababa, Ethiopia

**Keywords:** Governance, Economic growth, Universal health coverage, COVID-19, Infection rates, Fatality rates, Africa

## Abstract

**Background:**

The coronavirus disease 2019 (COVID-19) pandemic has disrupted lives across all countries and communities. It significantly reduced the global economic output and dealt health systems across the world a serious blow. There is growing evidence showing the progression of the COVID-19 pandemic and the impact it has on health systems, which should help to draw lessons for further consolidating and realizing universal health coverage (UHC) in all countries, complemented by more substantial government commitment and good governance, and continued full implementation of crucial policies and plans to avert COVID-19 and similar pandemic threats in the future. Therefore, the objective of the study was to assess the impact of good governance, economic growth and UHC on the COVID-19 infection rate and case fatality rate (CFR) among African countries.

**Methods:**

We employed an analytical ecological study design to assess the association between COVID-19 CFR and infection rate as dependent variables, and governance, economic development and UHC as independent variables. We extracted data from publicly available databases (i.e., Worldometer, Worldwide Governance Indicators, Our World in Data and WHO Global Health Observatory Repository). We employed a multivariable linear regression model to examine the association between the dependent variables and the set of explanatory variables. STATA version 14 software was used for data analysis.

**Results:**

All 54 African countries were covered by this study. The median observed COVID-19 CFR and infection rate were 1.65% and 233.46%, respectively. Results of multiple regression analysis for predicting COVID-19 infection rate indicated that COVID-19 government response stringency index (*β* = 0.038; 95% CI 0.001, 0.076; *P* = 0.046), per capita gross domestic product (GDP) (*β* = 0.514; 95% CI 0.158, 0.87; *P* = 0.006) and infectious disease components of UHC (*β* = 0.025; 95% CI 0.005, 0.045; *P* = 0.016) were associated with COVID-19 infection rates, while noncommunicable disease components of UHC (*β* = −0.064; 95% CI −0.114; −0.015; *P* = 0.012), prevalence of obesity among adults (*β* = 0.112; 95% CI 0.044; 0.18; *P* = 0.002) and per capita GDP (*β* = −0.918; 95% CI −1.583; −0.254; *P* = 0.008) were associated with COVID-19 CFR.

**Conclusions:**

The findings indicate that good governance practices, favourable economic indicators and UHC have a bearing on COVID-19 infection rate and CFR. Effective health system response through a primary healthcare approach and progressively taking measures to grow their economy and increase funding to the health sector to mitigate the risk of similar future pandemics would require African countries to move towards UHC, improve governance practices and ensure economic growth in order to reduce the impact of pandemics on populations.

**Supplementary Information:**

The online version contains supplementary material available at 10.1186/s12961-022-00932-0.

## Background

WHO first declared COVID-19 a public health emergency of international concern on 30 January 2020; on 11 March, it announced that the viral outbreak was officially a pandemic, the highest level of health emergency [1]. The novel coronavirus virus is highly contagious and has rapidly spread worldwide. The disease is causing a high death toll [2], with over 4.5 million new cases reported between 16 and 22 August 2021. Globally, the cumulative number of cases reported has exceeded 211 million, and the cumulative number of deaths was well over 4.4 million as of 24 August 2021 [3]. As of 11 August 2022, there have been 9 236 634 confirmed cases of COVID-19, including 17,409 deaths, reported for the WHO African Region. Considering the already increased disease burden and restricted capacity of health systems across countries in this region, the burden is likely greater than the numbers suggest [4, 5]. The development of evidence-based strategies is imperative to enable governments and healthcare systems, particularly those in low-income countries, to effectively deal with the evolving pandemic. While public health policies including non-pharmaceutical interventions (NPIs) to limit exposure and manage population risk have been reinstated several times in many locations in response to recurring resurgence of cases, governments continue to plan to return to normal economic and social life [6].

The pandemic has disrupted lives across all countries and communities and negatively affected global economic growth in 2020 and beyond, including in the African Region. Estimates indicate that the virus reduced global economic growth in 2020 to an annualized rate of −3.4% to −7.6%, with a recovery of 4.2–5.6% projected for 2021. Global trade is estimated to have fallen by 5.3% in 2020, despite being projected to grow by 8.0% in 2021 [7]. Understanding the factors associated with populations of similar size and structure having a higher risk for more widespread infection, severity of illness and mortality is critical [8, 9]. Higher population density may increase contact in the context of social distancing [8, 10]. Along with the pre-existing socioeconomic characteristics of the country, healthcare capacity and other health-related population features (i.e. smoking prevalence, obesity rate and global health indices) [6, 11–13], population health, population density, age demographics, delays in imposing national virus control measures, government containment policies, per capita gross domestic product (GDP) and climate may be factors contributing to disparities in outcomes across countries [13–16]. To curb the spread of the pandemic, NPI measures aim to reduce disease transmission both locally and globally and include bans on public gatherings, compulsory stay-at-home policies, mandated closures of schools and nonessential businesses, face mask ordinances, and quarantine and cordon sanitaire (i.e., a defined quarantine area from which those inside are not allowed to leave), among others [17, 18].

The emergence of the severe acute respiratory syndrome coronavirus 2 (SARS-CoV-2) and its subsequent spread has lived up to and surpassed many warnings and caused an evolving global public health and economic crisis [17]. In order to reverse/slow the progression of the pandemic, health systems must be resilient and must be able to continue providing primary services, even during the peak phases of COVID-19 waves including public health emergencies, through adherence to universal health coverage (UHC) principles and programmes, which can help prevent and adequately respond to disasters, minimizing their health and economic impact [19] and even building stronger health systems to strengthen healthcare delivery [2]. Strengthening health systems is the best way to safeguard against health system crises and collapse. Outbreaks are inevitable, but epidemics are not. Resilient health systems are our best defence at preventing disease outbreaks from becoming epidemics [2, 20, 21]. Evidence has shown that the COVID-19 experience should be an impetus towards achieving the goal of UHC in all countries, combined with more substantial governmental commitment and good governance that will aid in fully implementing this crucial set of policies and plans, drawing on the lessons of the COVID-19 pandemic, all of which underscore the fact that investing in health for all is not optional [2, 21, 22]. Hence, mounting cases and deaths have created a maximum public health and governance crisis in which the political and governmental structures in countries of the world have adopted curtailed travel and trade [18, 22]. Therefore, the objective of the study was to assess the impact of good governance, economic growth and UHC on the COVID-19 infection rate and case fatality rate (CFR) among African countries.

## Methods

### Study design and population

An analytical ecological study was designed to assess the impact of different indicators of governance, economics and development, and UHC on COVID-19-related indicators across Africa. Each country was used as an analysis unit. The 54 countries were countries of the African Region comprising Algeria, Angola, Benin, Botswana, Burkina Faso, Burundi, Cabo Verde, Cameroon, Central African Republic, Chad, Comoros, Congo, Côte d’Ivoire, Djibouti, Democratic Republic of the Congo, Egypt, Equatorial Guinea, Eritrea, Eswatini, Ethiopia, Gabon, Gambia, Ghana, Guinea, Guinea-Bissau, Kenya, Lesotho, Liberia, Libya, Madagascar, Malawi, Mali, Mauritania, Mauritius, Morocco, Mozambique, Namibia, Niger, Nigeria, Rwanda, Sao Tome and Principe, Senegal, Seychelles, Sierra Leone, Somalia, South Africa, South Sudan, Sudan, Togo, Tunisia, Uganda, United Republic of Tanzania, Zambia and Zimbabwe.

This study is a cross-sectional study considering multiple indices as variables. The method entailed data assembly from different sources through internet tools, using data openly available on official web pages of various organizations and collected for their purpose. The data sources for this analytical ecological study consisted primarily of international databases available from the Worldometer website (COVID cases up to 2019), Global Health Security (GHS) Index 2019, Our World in Data, WHO Global Health Observatory Repository (UHC service coverage index [SCI] 2017) and the World Bank World Development Indicators (WDI) and Worldwide Governance Indicators (WGI 2019). The confirmed cases of coronavirus and deaths are collected from Worldometer COVID Live update [23]. The study included only reported confirmed cases and deaths of COVID-19 and only from African countries. The daily number of confirmed cases and deaths for each country is reported and archived in the Worldometer. This is a cross-sectional study of the most recent 2020 data for African countries extracted from the WHO Global Health Observatory Repository [24]. The analysis considers countries as the study unit; hence, the study variables are the values for those countries.

### Study variables

The dependent variables in this study are the COVID-19 CFR and infection rate in each country as an indicator. These two measures represent the national-level COVID-19 public health issue well. The independent variable is the quality of government. As a proxy indicator for the quality of government, many studies have used the WGI provided by the World Bank. This database has been published by the World Bank since 1996 and consists of six sub-indicators/dimensions of governance: political stability and absence of violence/terrorism, corruption control, government effectiveness, regulatory quality, voice and accountability, and the rule of law (Additional file 1: Annex S1). All governance quality indicators have values ranging from −2.5 to 2.5; the higher the score, the higher the quality of government [25]. Predictor variables were divided into multiple categories: demographic and socioeconomic data such as population number and density, healthcare system such as expenditures and population density, the prevalence of obesity among adults and the stringency index describing the severity of the social distancing rules adopted by each country. The most recent available data were included in the analysis whenever a data series was reported by WHO and the World Bank (Additional file 1: Annex S1).

### Statistical analysis

The data collected in CSV (comma-separated values) format were exported into Microsoft Excel and then analysed with STATA version 14 software. The data were assessed for missing values, and a multiple imputation method was used to handle variables with missing data, taking the mean of the variable to impute within 5% of the observation. A normality test was conducted on the continuous numerical variable using histograms; categorical variables are presented as frequencies and percentages, and continuous variables are analysed descriptively using median, interquartile range (IQR), and minimum and maximum values. To reach normality, log transformation was used for the non-normal variables. Spearman correlation coefficients were estimated to examine the association between the COVID-19 CFR and infection rate and the selected variables. The risk factors associated with COVID-19 infection rate and CFR were determined by multivariable linear regression using an ordinary least-squares (OLS) regression model to study the association between the dependent variables and the set of explanatory variables. Univariable analysis was initially conducted using simple linear regression to identify each independent variable for inclusion in the multivariable analysis. Variables with a *P* value < 0.30 and those considered relevant were included in the multivariable analysis. The results are presented as unadjusted and adjusted regression coefficients (beta) (95% CI) and corresponding *P* values. *P* values are two-sided, with a 0.05 significance level.

## Results

A total of 54 African countries were covered in this study. Table 1 shows the means, standard deviations, minimums and maximums for the selected variables under consideration. The median observed COVID-19 CFR and infection rate were 1.65% and 233.46%, respectively. The median values for UHC SCI components for infectious and noncommunicable diseases were 44 and 67, respectively. Moreover, the median current health expenditure (% of GDP) in 2018 was 5.17; the median ambient and household air pollution-attributable death rate (per 100 000 population) was 82.71; the median prevalence of obesity among adults (body mass index [BMI] ≥ 30 kg/m^2^) was 8.2; the median per capita GDP (current USD) as of 2019 was $1337.6; the median government effectiveness score was −0.79 and the median COVID-19 government response stringency index was 47.9 (Table 1).

**Table 1:** Descriptive statistics of the variables

Variables	Obs	Mean	SD	Min	Max	Median	IQR
Observed COVID-19 CFR	54	2.16	1.45	0.15	7.52	1.65	1.96
COVID-19 infection rate	54	951.49	2 283.61	0.83	15 277.02	233.46	506.92
UHC SCI components: infectious diseases	54	44.72	14.30	11	78	44	18
UHC SCI components: noncommunicable diseases	54	66.82	5.57	52	81	67	5
Current health expenditure (% of GDP) 2018	53	5.65	2.57	2.14	16.06	5.17	2.85
Ambient and household air pollution-attributable death rate (per 100 000 population)	54	89.39	33.35	39.89	180.9	82.71	44.18
Prevalence of obesity among adults, BMI ≥ 30 kg/m^2^	54	10.529	7.061	3.6	31.8	8.2	5.9
Per capita GDP (current USD) 2019	54	2 555.56	3 171.32	126.9	17 448.3	1 337.6	2 506.8
Government effectiveness	54	−0.80	0.70	−2.45	0.87	−0.79	0.88
COVID-19 government response stringency index	54	47.9	18.01	11.11	80.56	47.9	25

*BMI* body mass index, *CFR* case fatality rate, *GDP* gross domestic product, *IQR* interquartile range, *SCI* UHC index of service coverage, *SD* standard deviation

The results of multiple regressions for predicting COVID-19 infection rates are presented in Table 2. Among the COVID-19-related factors, one additional unit increase in COVID-19 government response stringency index was associated with a 96.2% reduction in COVID-19 infection rate (*β* = 0.038; 95% CI 0.001, 0.076; *P* = 0.046). Among the economic-related factors, one additional unit increase in per capita GDP (current USD) was associated with a 48.6% reduction in COVID-19 infection rate (*β* = 0.514; 95% CI 0.158, 0.87; *P* = 0.006). And lastly, from the component of UHC, one additional unit increase in infectious disease components was associated with a 97.5% reduction in COVID-19 infection rate (*β* = 0.025; 95% CI 0.005, 0.045; *P* = 0.016).

**Table 2:** Multivariable linear regression analysis on predictors of COVID-19 infection rate and predictor variables among African countries

Variables	Coefficient	SE	*t*-value	*P*-value	[95% CI]		Significance
UHC SCI components: infectious diseases	0.025	0.01	2.50	0.016	0.005	0.045	**
Government effectiveness	0.017	0.264	0.06	0.949	−0.515	0.549	NS
Per capita GDP (current USD) 2019	0.514	0.177	2.91	0.006	0.158	0.87	***
COVID-19 government response stringency index	0.038	0.019	2.05	0.046	0.001	0.076	**
Prevalence of obesity among adults, BMI ≥ 30 kg/m^2^	0.017	0.022	0.76	0.453	−0.027	0.06	NS
Ambient and household air pollution-attributable death rate (per 100 000 population)	0.004	0.008	0.49	0.628	−0.012	0.02	NS
Mean dependent variable	5.580	SD dependent variable	1.611				
*R*-squared	0.610	No. observations	54				
*F*-test	12.187	Prob > *F*	0.000				
AIC	166.905	BIC	180.828				

*AIC* Akaike information criterion, *BIC* Bayesian information criterion, *BMI* body mass index, *CI* confidence interval, *GDP* gross domestic product, *NS* not significant, *SCI* UHC index of service coverage, *SE* standard error

***P < 0.01, **P < 0.05, *P < 0.1

***, ** and * respectively refers 1%, 5% and 10% level of confidence interval

The results of multiple regressions for predicting COVID-19 CFR are presented in Table 3. From the components of UHC, one additional unit increase in noncommunicable disease components was associated with a 93.6% increase in COVID-19 CFR (*β* = −0.064; 95% CI −0.114; −0.015; *P* = 0.012). Among the comorbidity-related factors, one additional unit increase in the prevalence of obesity among adults was associated with a 96.2% reduction in CFR (*β* = 0.112; 95% CI 0.044; 0.18; *P* = 0.002). Among the economic-related factors, one additional unit increase in per capita GDP was associated with an 8.2% increase in COVID-19 CFR (*β* = −0.918; 95% CI −1.583; −0.254; *P* = 0.008).

**Table 3:** Multivariable linear regression analysis on predictors of COVID-19 CFR and predictor variables among African countries

Variables	Coefficient	SE	*t*-value	*P*-value	[95% CI]		Significance
UHC SCI components: noncommunicable diseases	−0.064	0.025	−2.63	0.012	−0.114	−0.015	**
Prevalence of obesity among adults, BMI ≥ 30 kg/m^2^	0.112	0.034	3.32	0.002	0.044	0.18	***
Current health expenditure (% of GDP) 2018	−0.138	0.083	−1.65	0.106	−0.306	0.03	NS
Per capita GDP (current USD) 2019	−0.918	0.33	−2.78	0.008	−1.583	−0.254	***
Government effectiveness	−0.035	0.302	−0.11	0.909	−0.643	0.573	NS
Constant	12.723	3.815	3.33	0.002	5.048	20.398	***
Mean dependent variable	2.099	SD dependent variable	1.394				
*R*-squared	0.290	No. observations	53				
*F*-test	3.413	Prob > *F*	0.010				
AIC	178.500	BIC	190.322				

*AIC* Akaike information criterion, *BIC* Bayesian information criterion, *BMI* body mass index, *CI* confidence interval, *GDP* gross domestic product, *NS* not significant, *SCI* UHC index of service coverage, *SE* standard error

***P < 0.01, **P < 0.05, *P < 0.1

***, ** and * respectively refer to 1%, 5% and 10% level of confidence interval

## Discussion

The present study was conducted during a high wave of the pandemic from widely available public repository data to examine the impact of good governance, economic growth and UHC on COVID-19 infection rate and CFR in Africa, where the health system is weak and slow to improve, with high existing disease burden and fragile and ineffective health systems at the primary care level. The findings revealed that the infectious disease component of UHC SCI, per capita GDP and COVID-19 government response stringency index were factors associated with COVID-19 infection rates, and similarly, noncommunicable diseases of UHC SCI, the prevalence of obesity among adults and per capita GDP were factors associated with COVID-19 CFR among African countries.

In our study, countries with higher per capita GDP had higher reported COVID-19 infection rates. Similar to our finding, a statistically significant (*P* = 0.002) negative association was estimated between new COVID-19 cases and per capita GDP. In Europe, the country with the highest per capita GDP was found to experience the lowest change in new COVID-19 cases during the first wave of the pandemic, while the opposite was found for countries with lower per capita GDP [26]. Some evidence demonstrates that countries with higher GDP can provide their people with better public health programmes, all leading to enhanced prevention, treatment of disease, better health and longer life expectancy [27]. A study in low- and middle-income countries indicated that increasing GDP was negatively associated with all-cause, communicable and noncommunicable disease mortality in males and females across all age groups [28]. Interestingly, the opposite finding was documented for the association between economic growth and COVID-19 infection. One of the studies reported that the total number of COVID-19 infection cases per million population showed a mild negative correlation with the countries’ per capita GDP (purchasing power parity [PPP]) [29].

This finding is explained by the fact that higher-income countries showed a slightly higher number of cases per million population, which is likely due to the better availability of testing facilities in the high-income countries [29]. The CFR is much more important than the absolute number of infected persons, because most infections are asymptomatic or mildly symptomatic, and it is the mortality that we are more concerned about [29]. It was seen that the CFR of the various countries did not show any relation with the per capita GDP (PPP) [29]. In contrast, per capita GDP was associated with COVID-19 CFR in the countries/territories in the world with a proportion of people aged 65+ years larger than 15% [13], and socioeconomic factors such as per capita GDP were positively associated with COVID-19 CFR [15].

In addition, economic parameters might contribute equally well to shaping COVID-19 mortality. As the number of severe cases increases during the epidemic, the healthcare system can become overwhelmed and might be unable to receive and treat all those who need intensive care. However, seemingly contradicting this view, we also found that CFR was highest in countries with high per capita GDP and high total health expenditure as a share of GDP [5, 15]. Other research has demonstrated that in South-East Asia, public health expenditure alone contributes to improving life expectancy at birth, lower mortality among children under 5 years of age and lower noncommunicable disease mortality rates [30]. Exemplary of this is the Chinese government action that has significantly increased public health expenditure for epidemic governance, especially the spending on public health emergency treatment, government hospitals and major public health service projects, and has successfully controlled the epidemic [31, 32]. Other examples include Brazil, Russia, India, China and South Africa (BRICS nations) for the case of public health policies [33] as well as efficient implementation of public health policy [31, 34–36].

One of the factors more weakly associated with COVID-19 death rates was per capita GDP. Of many factors, only the role of per capita GDP was unexpected. A possible explanation for the association between higher levels of GDP and higher COVID-19 death rates may be more intra- and international travel in wealthier populations [14]. Even in high-income countries, the most affected populations are those from minority groups and of low socioeconomic background. For instance, the African American and Hispanic populations are more affected than the white groups in the United States [37, 38]. This point is further evidence of the need to integrate UHC principles in order to eliminate the health inequalities between different economic quintile groups of people in affluent countries [38]. However, evidence showed that a short-lived slowdown in real GDP growth took place during the COVID-19-induced lockdown and massive quarantines of large cities and intraregional travel [39]. Post-Cold War decades have witnessed accelerated real GDP growth across many low- and middle-income countries and emerging countries of the Global South [40]. Health financing mechanisms and the political economy of health spending continue to evolve rapidly in these vast regions [40].

In the past decades, European countries have experienced massive economic growth that has enabled them to invest in health and develop effective health systems that have brought several infections and diseases under control [26, 41, 42]. An examination of the COVID-19 response in several countries in Africa suggests that similar sustainable UHC strategies or crucial investments in healthcare systems are lacking [43]. COVID-19 appears to be a litmus test for the ability of national health systems to withstand health shocks while maintaining routine functions [43], and the pandemic is illustrating, albeit on a much larger stage, the lessons learned from past outbreaks: that resilience is an essential and cost-effective feature of a health system addressing complex challenges [44]. Health systems are facing widespread challenges, including changes in care delivery, escalating healthcare costs and the need to keep up with rapid scientific discoveries [45]. About 45% of the death toll in Africa and South-East Asia is attributable to infectious diseases, leading us to refocus our lens on global health diplomacy to strengthen their capacity for disease preparedness and response, which requires that they realign themselves and strengthen their health systems [46].

COVID-19 has amplified the urgency to accelerate efforts to build resilient and robust health systems and achieve progress towards UHC. Strong health systems with adequate resources are the key to successful crisis response and management. It has been demonstrated that countries with strong UHC, such as South Korea and Singapore, have outperformed during the COVID-19 pandemic [22, 44, 47]. As illustrated above, the combination and nexus of UHC with that of a resilient health system that can detect and respond to the pandemic provides a better platform to mitigate the pandemic effects. Hence, COVID-19 shows just how fragmented and underfunded health systems are worldwide. It is time for a radically reimagined approach to governance for global health [43]. The COVID-19 pandemic is the most significant public health emergency in a century. The national health system has been passive or has underestimated public health emergencies. Since the onset of the pandemic, every country's health system has been alert. They have started to review their health policies, programmes and resources [48], and recently the majority of countries have started to empower local health institutions and determine a proper time frame for strengthening the capacity of health systems by adopting innovative global approaches [22]. Hence, UHC is premised on having well-functioning health systems while responding to shocks from challenges such as COVID-19 [49]. In sum, the role of UHC can be more relevant when crises such as the COVID-19 infection occur [2].

The COVID-19 government response stringency index (describing the severity of the restrictions implemented by each country) was a factor associated with the COVID-19 infection rate. In line with most recent studies from 209 countries and territories, we did not observe a statistically significant association between the stringency index and COVID-19 CFR. However, subgroup analysis regarding testing policy in upper-income to middle-income and high-income countries indicated that if testing was ensured, an increase in the stringency index was associated with increased COVID-19 CFR [13]. In support of our finding, higher government stringency is a crucial predictor of the cumulative number of cases, where quick and early action by the government in imposing strict measures is essential in slowing or even reversing the growth rate of COVID-19 deaths and the spread of the virus [50–53], as the pandemic has upended healthcare, cultural, financial and government systems worldwide [54]. To strengthen our findings, many governments warn people to be particularly strict in following the recommended prevention measures, as COVID-19 may result in severe conditions requiring critical care including ventilation or death [55]. Moreover, this could explain the high trust in the government from a study that tracked the dynamic responses to the COVID-19 pandemic across 38 European countries and 621 regions. However, low confidence in the healthcare system is associated with higher adherence to social distancing policies and dramatically reduced mobility, suggesting a correlation for trust in the state concerning behavioural responses during a crisis [56] as well as decreases in mobility, which were approximately linearly related to subsequent decreases in mobility and a relative decline in COVID-19 case growth rate [57]. In line with this, other studies documented moderate evidence suggesting that countries with a democratic regime were those with the highest CFR and stringency index, showing that the highest CFR values were reached for intermediate values of the stringency index [15].

Similar to our result, other studies have reported that effective governance is one of the factors associated with the COVID-19 infection rate. Evidence showed that the implementation of less strict intervention measures was ineffective in reducing the number of deaths, whereas interventions at higher levels of severity reduced deaths [50]. Using daily data for 32 countries, it was found that the greater the strength of government interventions at an early stage, the more effective these were in slowing or reversing the growth rate of deaths. These results can inform governments in responding to future COVID-19 outbreaks or other pandemics, not least because there is a possibility of further waves of COVID-19 infections and deaths as governments progressively relax their interventions [53]. Likewise, in Mediterranean countries, empirical evidence on the effectiveness of the governments’ policy measures considering the categories of response (lockdowns, social distancing, movement restrictions, public health measures, and governance and socioeconomic measures) in response to the COVID-19 pandemic showed that the earlier that governments act concerning the evolution of the epidemic, the lower the total cumulative incidence due to the epidemic wave [58].

Similar to our finding, other studies have highlighted that obesity is associated with increased risk of COVID-19 [59, 60]. Along the same lines, for COVID-19 patients, even if different across countries, obesity was one of the main risk factors associated with hospitalization and the critical evolution of the disease [11, 61–65]. Compared with nonobese patients, obese patients had a significantly increased risk of infection. Clinically severe disease and mortality [11, 62, 64–66] and obesity may be clinical predictors for adverse outcomes [14, 63]. The explanation for this is the disproportionate impact of COVID-19 on pulmonary function in patients with obesity and severe obesity, in which obesity is associated with decreased expiratory reserve volume, functional capacity and respiratory system compliance. Furthermore, increased inflammatory cytokines associated with obesity may contribute to the increased morbidity associated with obesity in COVID-19 infections [65].

Our study has some limitation to be considered. Because of the ecological study design, it is difficult to make a clear association between dependent and explanatory variables to draw conclusions, as the data are used in aggregate rather than for individual patients. We did not include some of the most important variables such as climate change or biological indicators (like underlying genetic factors) in the study. Additionally, the data for the study are from different sources, and we did not expect a perfect or complete dataset and do not disaggregate by important parameters. The true number of COVID-19 cases and deaths is likely undetected or underreported in most of the countries.

In conclusion, our study indicates that there is an association between the COVID-19 infection rate and COVID-19 government response stringency index, per capita GDP and components of the UHC index of service coverage indicator for infectious diseases, while components of the UHC index of service coverage indicator for noncommunicable diseases, prevalence of obesity among adults and per capita GDP were found to be associated with the COVID-19 CFR. African governments should boost their efforts to support UHC through greater commitment to the principles of primary healthcare, dedicating more resources to the health sector, and implementing and monitoring stringent NPIs to minimize the risk of new infections in order to maintain public health policies across countries. Our study is yet further evidence for the need to renew African states’ promises to strengthen essential primary healthcare services that include preventive and promotive healthcare to prevent and reduce chronic diseases in order to reduce the risk of infection and mortality from COVID-19.

## Supplementary Information

**Additional file 1: Annex S1.** Explanatory variables, descriptions and sources.

## Data Availability

The data analysed during this study are included in this article and are available from the corresponding author upon reasonable request.
