# Supplementary material for: Impact of good governance, economic growth and universal health coverage on COVID-19 infection and case fatality rates in Africa
Source: Health Res Policy Syst. 2022 Nov 28;20:130. doi: 10.1186/s12961-022-00932-0 (PMC9702649; doi:10.1186/s12961-022-00932-0)
Supplement: Supplementary file 1 — Additional file 1: Annex S1. Explanatory variables, descriptions and sources. [file 12961_2022_932_MOESM1_ESM.doc]

**Annex S**1: Explanatory variables, descriptions and sources

| Explanatory variables | Definitions | Source of data |
| --- | --- | --- |
| COVID-19 Government Response Stringency Index (Stringency Index) | This composite measure is a simple additive score of nine indicators measured on an ordinal scale, rescaled to vary from 0 to 100. The specific policy and response categories are coded as follows: school closures; workplace closures; cancel public events; restrictions on gatherings; close public transport; public information campaigns; stay at home; restrictions on internal movement; international travel controls; testing policy; contract tracing; face coverings and vaccination policy. | Our World in Data |
| Ambient and household air pollution attributable death rate (per 100,000 population) | The mortality attributable to the joint effects of household and ambient air pollution can be expressed as : Number of deaths Death rate Death rates are calculated by dividing the number of deaths by the total population (or indicated if a different population group is used, e.g. children under 5 years). Evidence from epidemiological studies have shown that exposure to air pollution is linked, among others, to the important diseases taken into account in this estimate: Acute respiratory infections (estimated for all ages ); Cerebrovascular diseases in adults (estimated above 25 years); Ischaemic heart diseases in adults (estimated above 25 years); Chronic obstructive pulmonary disease in adults (estimated above 25 years); and Lung cancer in adults (estimated above 25 years). | WHO Global Health Observatory indicator |
| Governance | Traditions and institutions by which authority in a country is exercised. This includes the process by which governments are selected, monitored and replaced; the capacity of the government to effectively formulate and implement sound policies; and the respect of citizens and the state for the institutions that govern economic and social interactions among them. | World Bank |
| Voice and Accountability (VA) | Capturing perceptions of the extent to which a country's citizens are able to participate in selecting their government, as well as freedom of expression, freedom of association, and a free media. | The Worldwide Governance Indicators |
| Political Stability and Absence of Violence/Terrorism (PV) | Capturing perceptions of the likelihood that the government will be destabilized or overthrown by unconstitutional or violent means, including politically-motivated violence and terrorism | The Worldwide Governance Indicators |
| The capacity of the government to effectively formulate and implement sound policies: | | |
| Government Effectiveness (GE) | Capturing perceptions of the quality of public services, the quality of the civil service and the degree of its independence from political pressures, the quality of policy formulation and implementation, and the credibility of the government's commitment to such policies. | The Worldwide Governance Indicators |
| Regulatory Quality (RQ) | Capturing perceptions of the ability of the government to formulate and implement sound policies and regulations that permit and promote private sector development. | The Worldwide Governance Indicators |
| The respect of citizens and the state for the institutions that govern economic and social interactions among them: | | |
| Rule of Law (RL) | Capturing perceptions of the extent to which agents have confidence in and abide by the rules of society, and in particular the quality of contract enforcement, property rights, the police, and the courts, as well as the likelihood of crime and violence. | The Worldwide Governance Indicators |
| Control of Corruption (CC) | Capturing perceptions of the extent to which public power is exercised for private gain, including both petty and grand forms of corruption, as well as "capture" of the state by elites and private interests | The Worldwide Governance Indicators |
| Universal health coverage (UHC) | Universal health coverage means that all people have access to the health services they need, when and where they need them, without financial hardship. It includes the full range of essential health services, from health promotion to prevention, treatment, rehabilitation, and palliative care. | WHO |
| Universal health coverage (UHC) Service coverage indicators (SCI) components: Service capacity and access | Coverage of essential health services (defined as the average coverage of essential services based on tracer interventions that include basic hospital access; health worker density; access to essential medicines and compliance with the international health regulations, among the general and the most disadvantaged population). | WHO Global Health Observatory indicator |
| UHC SCI components: Noncommunicable diseases | Coverage of essential health services (defined as the average coverage of essential services based on tracer interventions that Includes prevalence of raised blood pressure; mean fasting plasma glucose; cervical cancer screening; tobacco control and best-practice policy implemented for industrially produced trans-fatty acids (TFA), among the general and the most disadvantaged population). | WHO Global Health Observatory indicator |
| UHC SCI components: Reproductive, maternal, newborn and child health | Coverage of essential health services (defined as the average coverage of essential services based on tracer interventions that covers family planning; antenatal care; full child immunization and health-seeking behaviour for child illness  , among the general and the most disadvantaged population). | WHO Global Health Observatory indicator |
| UHC SCI components: Infectious diseases | Coverage of essential health services (defined as the average coverage of essential services based on tracer interventions that comprise of tuberculosis effective treatment; HIV antiretroviral treatment; insecticide-treated nets coverage for malaria prevention and adequate sanitation, among the general and the most disadvantaged population). | WHO Global Health Observatory indicator |
| Current health expenditure (CHE) as percentage of gross domestic product (GDP) (%) | Health spending measures the final consumption of health care goods and services (i.e. current health expenditure) including personal health care (curative care, rehabilitative care, long-term care, ancillary services and medical goods) and collective services (prevention and public health services as well as health administration), but excluding spending on investments. Health care is financed through a mix of financing arrangements including government spending and compulsory health insurance. | World Health Organization Global Health Expenditure database |
| GDP per capita (current US$) | Gross domestic product (GDP) is the standard measure of the value added created through the production of goods and services in a country during a certain period. As such, it also measures the income earned from that production, or the total amount spent on final goods and services (less imports). | World Bank national accounts data, and OECD National Accounts data files |
| Prevalence of obesity among adults | Percentage of defined population with a body mass index (BMI) of 30 kg/m2 or higher among adults | WHO Global Health Observatory indicator |
